# The impact of Toll-like-receptor-9 on intestinal microbiota composition and extra-intestinal sequelae in experimental *Toxoplasma gondii* induced ileitis

**DOI:** 10.1186/1757-4749-6-19

**Published:** 2014-06-06

**Authors:** Stefan Bereswill, Anja A Kühl, Marie Alutis, André Fischer, Luisa Möhle, Daniela Struck, Oliver Liesenfeld, Ulf B Göbel, Ildikò R Dunay, Markus M Heimesaat

**Affiliations:** 1 Department of Microbiology and Hygiene, Charité - University Medicine Berlin, Centrum 5, Campus Benjamin Franklin, Hindenburgdamm 27, D-12203 Berlin, Germany; 2 Department of Internal Medicine, Rheumatology and Clinical Immunology/Research Center ImmunoSciences (RCIS), Charité - University Medicine Berlin, Berlin, Germany; 3 Department of Microbiology and Hygiene, University of Magdeburg, Magdeburg, Germany

**Keywords:** *Toxoplasma gondii*, Acute ileitis, TLR-9, Th1-type immunopathology, Extra-intestinal immune responses, Intracerebral inflammation, Pro-inflammatory cytokines, Intestinal microbiota composition, Bacterial translocation, Systemic inflammatory response, Bifidobacteria, FOXP3, Regulatory T cells, Gut-brain-axis

## Abstract

**Background:**

Following peroral *Toxoplasma (T.) gondii* infection, susceptible mice develop acute ileitis due to a microbiota-dependent Th1 type immunopathology. Toll-like-receptor (TLR)-9 is known to recognize bacterial DNA and mediates intestinal inflammation, but its impact on intestinal microbiota composition and extra-intestinal sequelae following *T. gondii* infection has not yet been elucidated.

**Methods and results:**

Seven days following peroral infection (p.i.) with 100 cysts of *T. gondii* ME49 strain, TLR-9^-/-^ and wildtype (WT) mice suffered from comparable ileitis, whereas ileal parasitic loads as well as IFN-γ and nitric oxide levels were higher in TLR-9^-/-^ compared to WT mice. Locally, TLR-9^-/-^ mice exhibited increased ileal CD3+, but not FOXP3+ cell numbers at day 7 p.i.; in mesenteric lymph nodes IFN-γ-producing CD4+ cell numbers and TNF-α and IFN-γ concentrations were also increased in TLR-9^-/-^ compared to WT mice. *T. gondii* DNA levels, however, did not differ in mice of either genotype. Differences in intestinal microbiota were rather subtle except for bifidobacteria that were virtually absent in both, naïve and *T. gondii* infected TLR-9^-/-^, but not WT mice. Extra-intestinally, TLR-9^-/-^ mice displayed less distinct systemic immune responses as indicated by lower serum IL-6, and splenic TNF-α and IFN-γ levels as compared to WT mice despite higher translocation rates of intestinal bacteria to extra-intestinal compartments such as liver, spleen, kidney, and cardiac blood. Most importantly, brains were also affected in this inflammatory scenario as early as day 7 p.i. Remarkably, TLR-9^-/-^ mice exhibited more pronounced inflammatory infiltrates with higher numbers of F4/80+ macrophages and microglia in the cortex and meninges as compared to WT mice, whereas *T. gondii* DNA levels did not differ.

**Conclusion:**

We here show that TLR-9 is not required for the development of *T. gondii* induced ileitis but mediates distinct inflammatory changes in intestinal and extra-intestinal compartments including the brain.

## Introduction

Seven days following peroral infection with 100 cysts of *Toxoplasma (T.) gondii* ME49 strain, susceptible mice develop massive necrosis in the terminal ileum and succumb to the infection [1]. This fatal scenario is due to a classical Th1-type immunopathology orchestrated by intestinal epithelial cells, granulocytes, macrophages, monocytes, dendritic cells, and lymphocytes [2]. Early upon *T. gondii* infection, parasitic interaction with antigen presenting cells results in activation of CD4+ T cells and over-production of mediators such as IFN-γ, TNF-α, nitric oxide (NO), IL-6, and MCP-1 among others comprising a “pro-inflammatory cytokine storm” [3-7]. Hence, the underlying immunopathogenesis resembles key features of chronic inflammatory bowel diseases (IBD) such as Crohn’s disease in the acute stage [2,8]. Furthermore, we recently showed that lipopolysaccharide (LPS) derived from the intestinal microbiota mediates *T. gondii* induced immunopathology via Toll-like-receptor (TLR) -4 signaling [9-11].

Bacterial unmethylated CpG DNA is the classical ligand for TLR-9 signalling [12-14]. Upon binding, transcription factors such as NF-κB, IFN regulatory factor-7, and AP-1 among others become activated in a MyD88-dependent fashion leading to Th1 type immune responses [15,16]. Initially, TLR-9 was shown to be crucial for an effective Th1-type immune response following oral *T. gondii* infection of mice [17]. However, the direct recognition of *T. gondii* molecules through TLR-9 is controversially discussed [18]. Parasitic DNA and RNA have been shown to activate innate immune responses via TLR-7 and TLR-9, but mice lacking TLR-9 alone were not susceptible to *T. gondii* infection [19]. In addition, TLR-11 and TLR-12 acting as heterodimers were shown to be essentially required for sensing of *Toxoplasma* profilin [19].

Given that bacterial DNA derived from the commensal microbiota provide pivotal immune-stimulatory molecules for effective host defense against parasitic infection [20], the distinct microbiota composition displays an essential determinant for the immunopathogenesis in murine *T. gondii* induced acute ileitis. In the present study we performed a comprehensive survey of quantitative and qualitative changes in the intestinal microbiota composition of TLR-9^-/-^ and WT control mice following ileitis induction. Furthermore, we assessed immunopathological sequelae following peroral *T. gondii* infection in intestinal as well as extra-intestinal compartments such as spleen, liver, kidneys, and brain.

## Results

### Acute ileal immunopathology in TLR-9^-/-^ mice following peroral *T. gondii* infection

**Figure 1:**
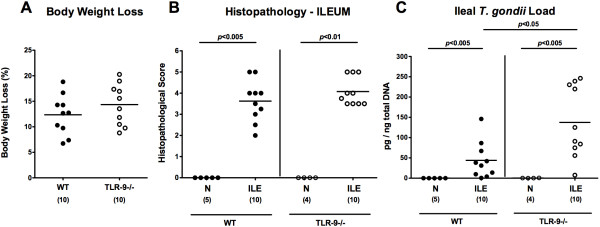
**Clinical conditions and small intestinal histopathology following ileitis induction upon peroral *T. gondii *infection.** In order to induce acute ileitis, C57BL/6 wildtype (WT; black circles) and TLR-9 deficient (TLR-9-/-; white circles) mice were perorally infected with 100 cysts of *T. gondii* ME49 strain on day 0. **(A)** Relative loss of body weights between day 7 following ileitis induction (ILE) and day 0 (naïve mice; N) were determined (in %). Furthermore, **(B)** histopathological mucosal changes were assessed in ileal paraffin sections from respective mice seven days following peroral *T. gondii* infection applying a standardized histopathological score. **(C)***T. gondii* DNA was determined in *ex vivo* ileal biopsies at day 7 p.i.. Naïve mice served as negative controls (N). Numbers of analyzed mice are given in parentheses. Medians (black bars) and significance levels (*P-*values) determined by Mann–Whitney-*U* test are indicated. Data shown are pooled from three independent experiments.

In order to induce acute ileitis TLR-9^-/-^ and wildtype (WT) mice were subjected to a peroral infection with 100 cysts of *T. gondii* ME49 strain. Seven days thereafter mice of either genotype were compromised due to wasting disease and suffered from comparable relative body weight losses (Figure 1A). In addition, TLR-9^-/-^ and WT mice displayed similar severity of acute ileitis as indicated by comparable ileal histopathological scores (Figure 1B). Notably, TLR-9^-/-^ mice harbored significantly higher parasitic DNA loads in the small intestines as compared to WT controls at day 7 p.i. (p < 0.05; Figure 1C).

**Figure 2:**
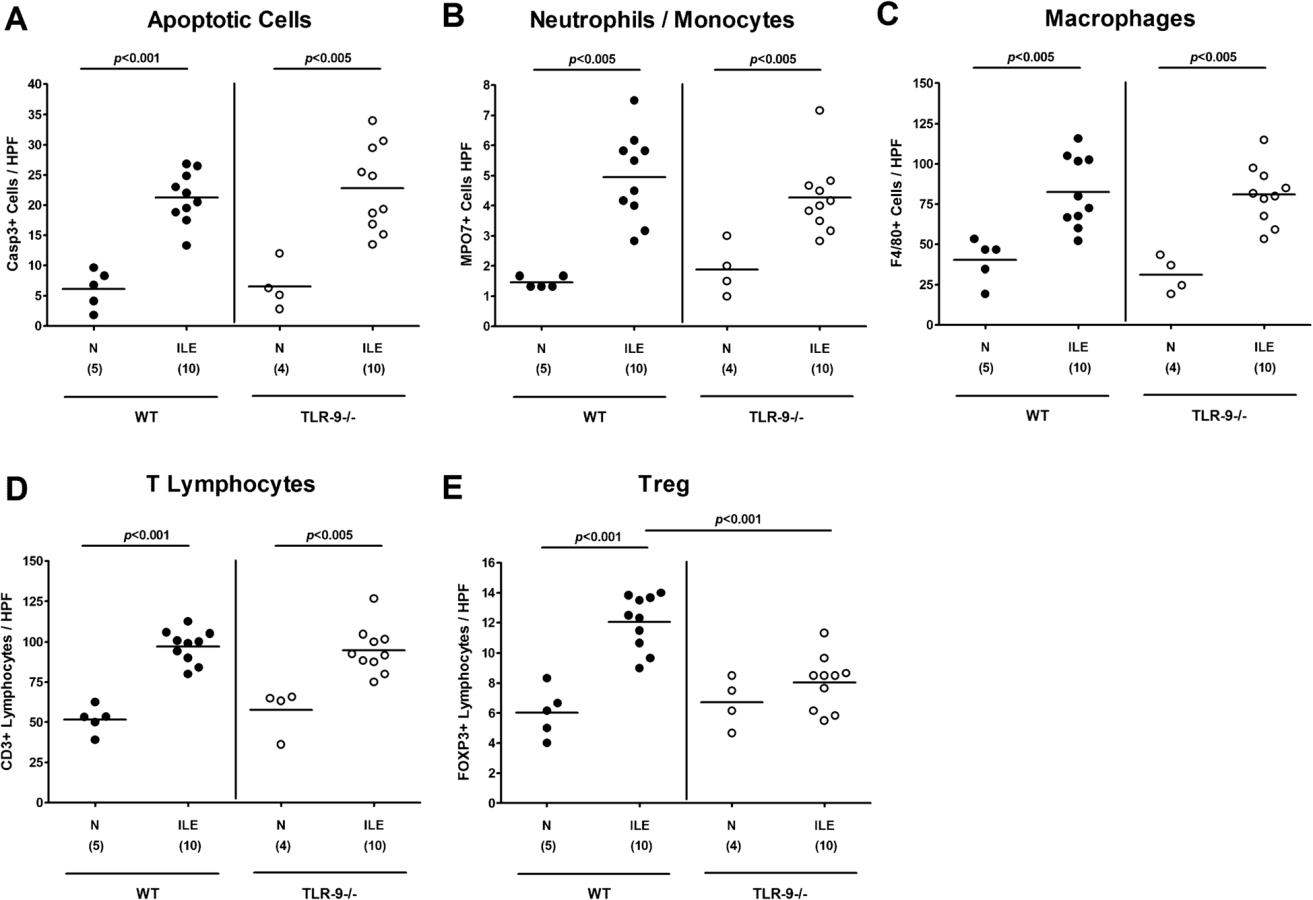
**Small intestinal pro-inflammatory immune cell responses following ileitis induction.** Pro-inflammatory immune cell responses were assessed in ileal paraffin sections derived from C57BL/6 wildtype (WT; black circles) and TLR-9 deficient (TLR-9-/-; white circles) mice seven days following ileitis induction (ILE) applying *in situ* immunohistochemistry. The average numbers of ileal **(A)** apoptotic cells (positive for caspase-3; Casp3), **(B)** neutrophilic granulocytes and monocytes (positive for MPO-7), **(C)** macrophages (positive for F4/80), **(D)** T lymphocytes (positive for CD3), and **(E)** regulatory T cells (Treg, positive for FOXP3) were determined in six high power fields (HPF, 400 × magnification) per animal by light microscopy. Naïve mice served as negative controls (N). Numbers of analyzed mice (in parentheses), means (black bars) and levels of significance (*P*-values) as compared to the respective groups (determined by Mann–Whitney-*U* test) are indicated. Data shown were pooled from three independent experiments.

We next quantitatively assessed ileum mucosal apoptotic cells as well as the influx of distinct immune cell populations into the small intestinal mucosa and lamina propria during acute ileitis applying *in situ* immunohistochemical stainings of ileal paraffin sections. At day 7 p.i., numbers of caspase-3+ apoptotic cells increased to comparable levels in ilea of TLR-9^-/-^ and WT mice (Figure 2A), thus further underlining the clinical and histopathological results. In addition, numbers of T cells, the major driving forces of *T. gondii* induced acute ileitis, as well as of neutrophilic granulocytes, monocytes, and macrophages exerting oxidative stress to the intestinal epithelium increased comparably in TLR-9^-/-^ and WT mice until day 7 p.i. (Figure 2B-D). Interestingly, FOXP3+ regulatory T cell (Treg) numbers increased in WT, but not TLR-9^-/-^ mice upon ileitis induction (p < 0.001; Figure 2E). Taken together, TLR-9^-/-^ mice were not protected from *T. gondii* induced acute ileitis and were unable to control replication of the parasite.

**Figure 3:**
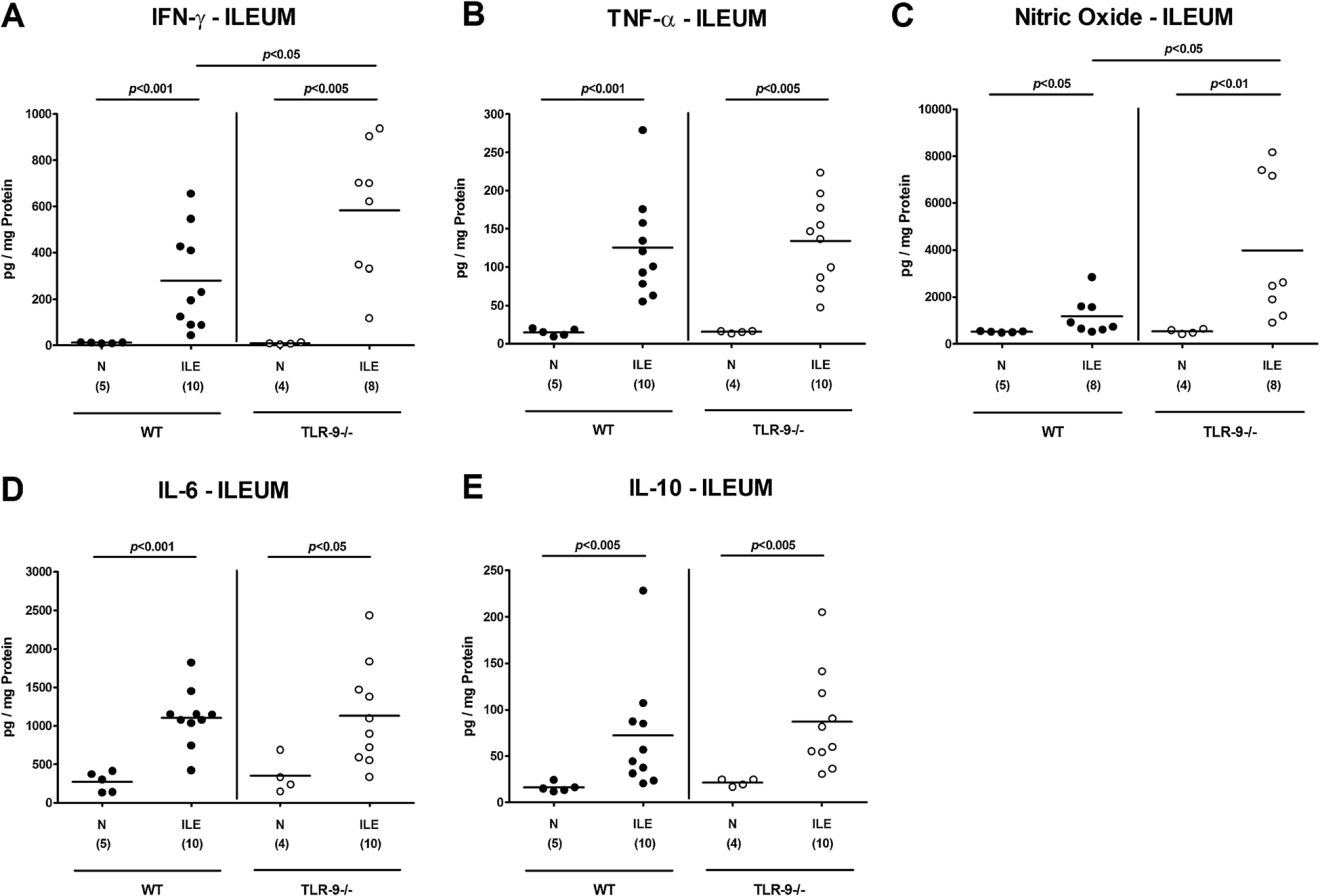
**Ileal cytokine secretion following ileitis induction. (A)** IFN-γ, **(B)** TNF-α, **(C)** nitric oxide, **(D)** IL-6, and **(E)** IL-10 protein levels were determined in *ex vivo* ileal biopsies derived from C57BL/6 wildtype (WT; black circles) and TLR-9 deficient (TLR-9-/-; white circles) mice seven days following ileitis induction (ILE) as described in methods. Naïve mice served as negative controls (N). Numbers of analyzed mice (in parentheses), means (black bars) and levels of significance (*P*-values) as compared to the respective groups (determined by Mann–Whitney-*U* test) are indicated. Data shown were pooled from three independent experiments.

**Figure 4:**
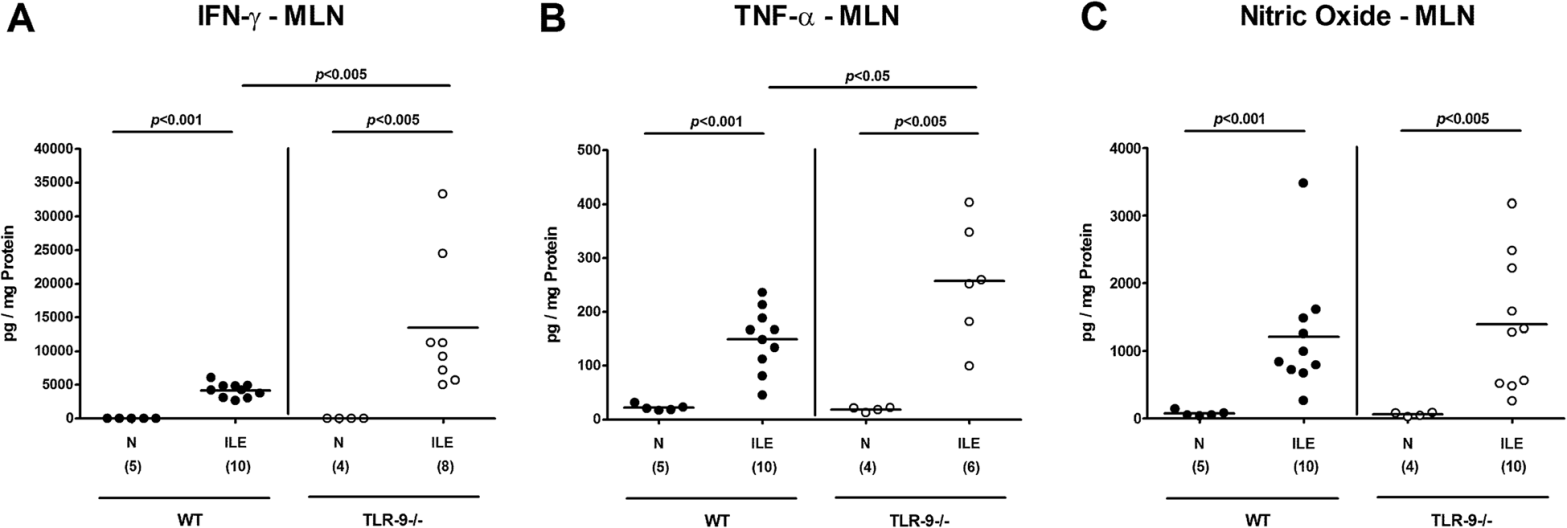
**Pro-inflammatory cytokine responses in mesenteric lymph nodes following ileitis induction. (A)** IFN-γ, **B)** TNF-α, and **(C)** nitric oxide protein concentrations were determined in *ex vivo* biopsies of mesenteric lymph nodes (MLN) derived from C57BL/6 wildtype (WT; black circles) and TLR-9 deficient (TLR-9-/-; white circles) mice seven days following ileitis induction (ILE). Naïve mice served as negative controls (N). Numbers of analyzed mice (in parentheses), means (black bars) and levels of significance (*P*-values) as compared to the respective groups (determined by Mann–Whitney-*U* test) are indicated. Data shown were pooled from three independent experiments.

**Figure 5:**
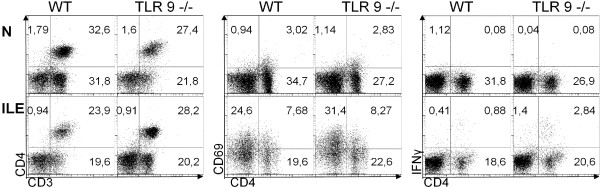
**Flow cytometry analysis of lymphocyte populations in mesenteric lymph nodes following ileitis induction.** Lymphocyte populations were isolated from mesenteric lymph nodes (MLN) derived from C57BL/6 wildtype (WT) and TLR-9 deficient (TLR-9-/-) mice before (naïve) and seven days following ileitis induction (ILE) and analyzed by flow cytometry. Frequencies of CD4+ cells producing IFN-γ (**right panel**), CD4+ CD3+ cells (**left panel**), as well as CD69+ CD4+ cells (**middle panel**), are indicated in representative FACS plots (in %). Data shown are representative for three independent experiments.

We next determined pro-inflammatory cytokine levels in *ex vivo* ileal biopsies taken before and seven days after ileitis induction. Until day 7 p.i., intestinal IFN-γ, TNF-α, nitric oxide (NO), and IL-6 levels had increased in mice of either genotype (p < 0.05-0.001; Figure 3). TLR-9^-/-^ mice, however, displayed even higher ileal IFN-γ and NO concentrations as compared to WT controls at day 7 p.i. (p < 0.05; Figure 3A,C). Notably, small intestinal concentrations of the anti-inflammatory cytokine IL-10 essentially involved in counteracting *T. gondii* induced immunopathology [21] increased to comparable levels in mice of either genotype until day 7 p.i. (Figure 3E). In the following we assessed pro-inflammatory cytokine responses in mesenteric lymph nodes (MLNs) draining the small intestinal tract. Again, IFN-γ, TNF-α, and NO protein levels increased multifold upon *T. gondii* infection (p < 0.005-0.001; Figure 4A-C), but the former two cytokines were significantly higher in TLR-9^-/-^ mice as compared to WT controls (p < 0.005 and p < 0.05, respectively; Figure 4A,B). Furthermore, flow cytometry analysis of lymphocytes isolated from MLNs revealed a higher abundance of IFN-γ producing CD4+ cells in TLR-9^-/-^ versus WT mice at day 7 p.i. (2.84% versus 0.88%; Figure 5), whereas naïve mice of either genotype exhibited similar frequencies of CD4+ IFN-γ + cells. Furthermore, relative frequencies of CD3+ CD4+ cells were comparable in MLNs of naïve and *T. gondii* infected mice of either genotype, whereas percentages of activated T lymphocytes (i.e. CD69+ CD4+ cells) increased comparably upon *T. gondii* infection in both, TLR-9^-/-^ and WT animals (Figure 5).

### Intestinal microbiota changes and bacterial translocation to extra-intestinal compartments in TLR-9^-/-^ mice with acute ileitis

**Figure 6:**
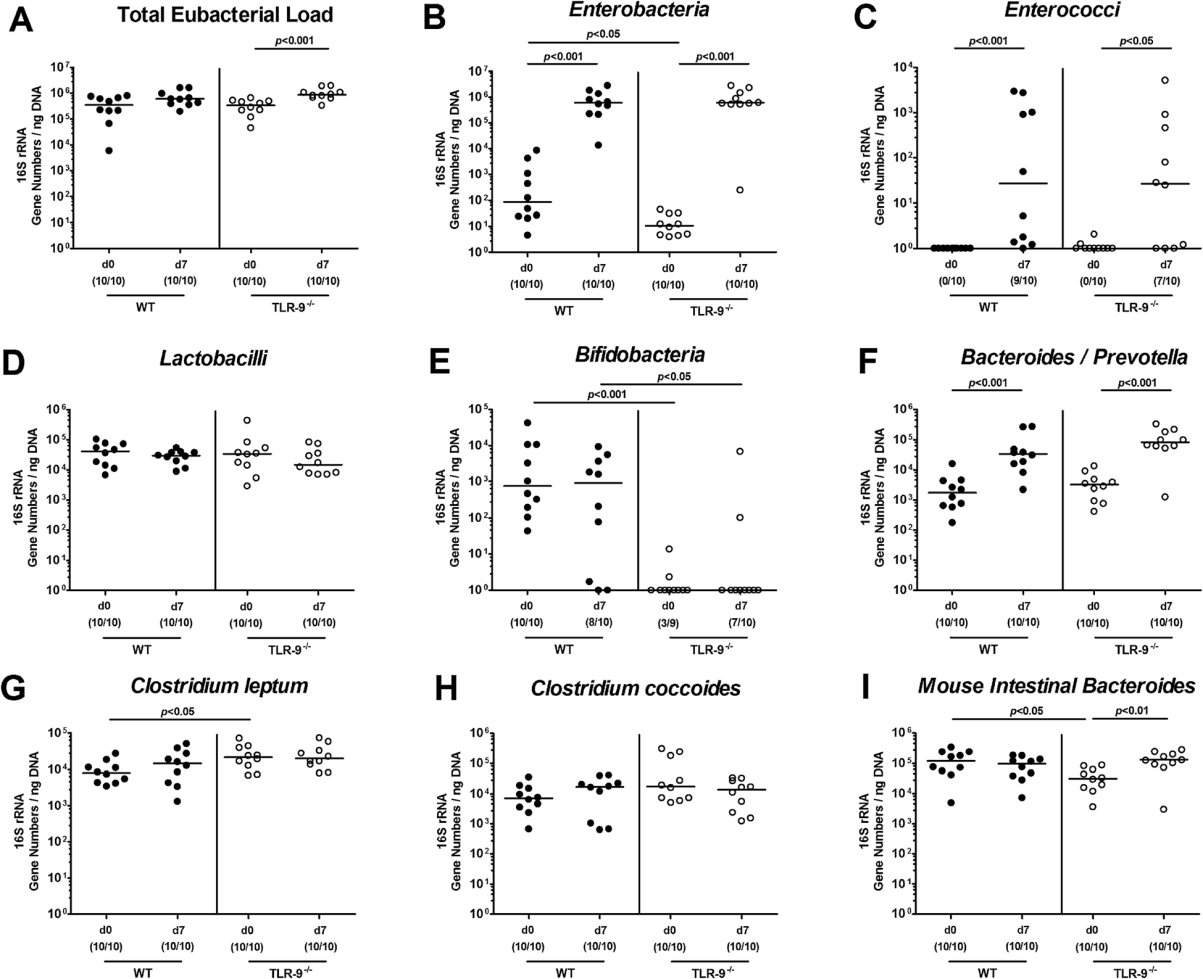
**Intestinal microbiota composition of mice following ileitis induction.** Main intestinal bacterial groups were quantified by molecular analysis of fecal samples derived from C57BL/6 wildtype (WT; black circles) and TLR-9 deficient (TLR-9-/-; white circles) mice before (N, naïve) and seven days after ileitis induction by peroral *T. gondii* infection (ILE). Quantitative real time-PCR analyses amplified bacterial 16S rRNA variable regions and 16S rRNA gene numbers/ng DNA from the following bacterial groups: **(A)** Total eubacterial load, **(B)***Enterobacteria*, **(C)***Enterococci*, **(D)***Lactobacilli*, **(E)***Bifidobacteria*, **(F)***Bacteroides/Prevotella* spp, **(G)***Clostridium leptum* group, **(H)***Clostridium coccoides* group, and **(I)***Mouse intestinal Bacteroides.* Numbers of mice harboring the respective bacterial 16S rRNA out of the total number of analyzed animals are given in parentheses. Medians and significance levels (*P*-values) determined by Mann–Whitney-*U* test are indicated. Data shown were pooled from three independent experiments.

Given that the commensal intestinal microbiota composition mediates induction and perpetuation of intestinal immunopathogenesis [2,9,10,22], we next performed a comprehensive quantitative molecular survey of the main fecal bacterial groups derived from TLR-9^-/-^ and WT mice, before and seven days after ileitis induction. Until day 7 p.i., the total eubacterial load increased slightly, but only reached statistical significance in TLR-9^-/-^ mice (p < 0.001; Figure 6A). Higher total bacterial loads at day 7 as compared to day 0 were due to increases in enterobacteria, enterococci, *Bacteroides/Prevotella* spp. loads in both, WT and TLR-9^-/-^ mice (Figure 6B,C,F), and of Mouse Intestinal *Bacteroides* in the latter (Figure 6I). Notably, naive TLR-9^-/-^ mice harbored slightly lower fecal enterobacteria and Mouse Intestinal *Bacteroides* DNA, whereas *Clostridium leptum* loads were higher compared to naïve WT mice (Figure 6B, I, G), Remarkably, bifidobacteria were virtually absent in TLR-9^-/-^, but not WT mice at either time point (Figure 6E).

**Figure 7:**
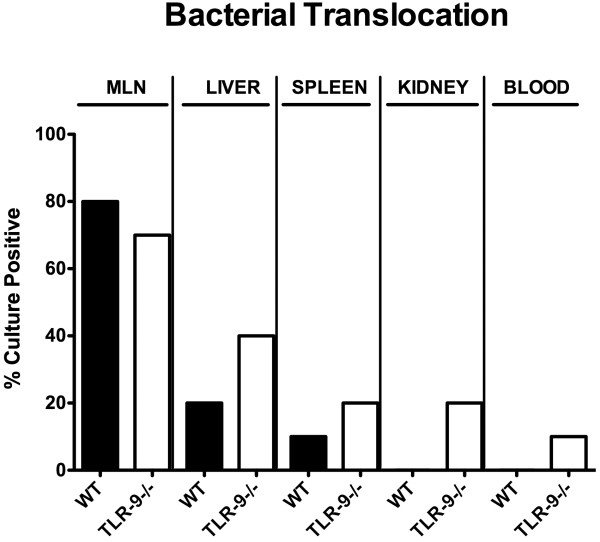
**Bacterial translocation following ileitis induction.** Relative translocation frequencies (%) of live bacteria were determined in *ex vivo* biopsies of mesenteric lymph nodes (MLN), liver, spleen, kidneys, and cardiac blood derived from C57BL/6 wildtype (WT; black bars; n = 10) and TLR-9 deficient (TLR-9-/-; white bars; n = 10) mice by culture in enrichment broths seven days after ileitis induction. Data shown were pooled from three independent experiments.

Given that a compromised epithelial barrier facilitates bacterial translocation from the intestinal lumen to sub-epithelial and further extra-intestinal sites, we next assessed translocation rates of viable bacteria. Of interest, whereas commensal intestinal bacteria could be cultured from MLNs of TLR-9^-/-^ and WT mice at identical frequencies (80%), mean translocation rates to spleen (20% vs 10%, respectively), liver (40% vs 20%, respectively), and kidneys (20% vs 10%, respectively) were higher in TLR-9^-/-^ as compared to WT mice (Figure 7). Furthermore, even 10% of cardiac blood samples derived from TLR-9^-/-^ mice were culture-positive whereas all blood cultures were negative in WT animals at day 7 p.i. (Figure 7). Cultured translocated bacteria comprised commensal intestinal species such as *E. coli*, *Enterococcus* spp. and *Lactobacillus* spp., but no obligate anaerobic bacteria (not shown). Notably, respective translocation frequencies were identical in independent experiments. Taken together, these data point towards a more severe damage to the ileal epithelial barrier in diseased TLR-9^-/-^ mice as compared to WT controls, thus facilitating translocation of live commensal bacteria originating from the intestinal lumen to extra-intestinal compartments.

### Extra-intestinal immunopathology in TLR-9^-/-^ mice with acute ileitis

**Figure 8:**
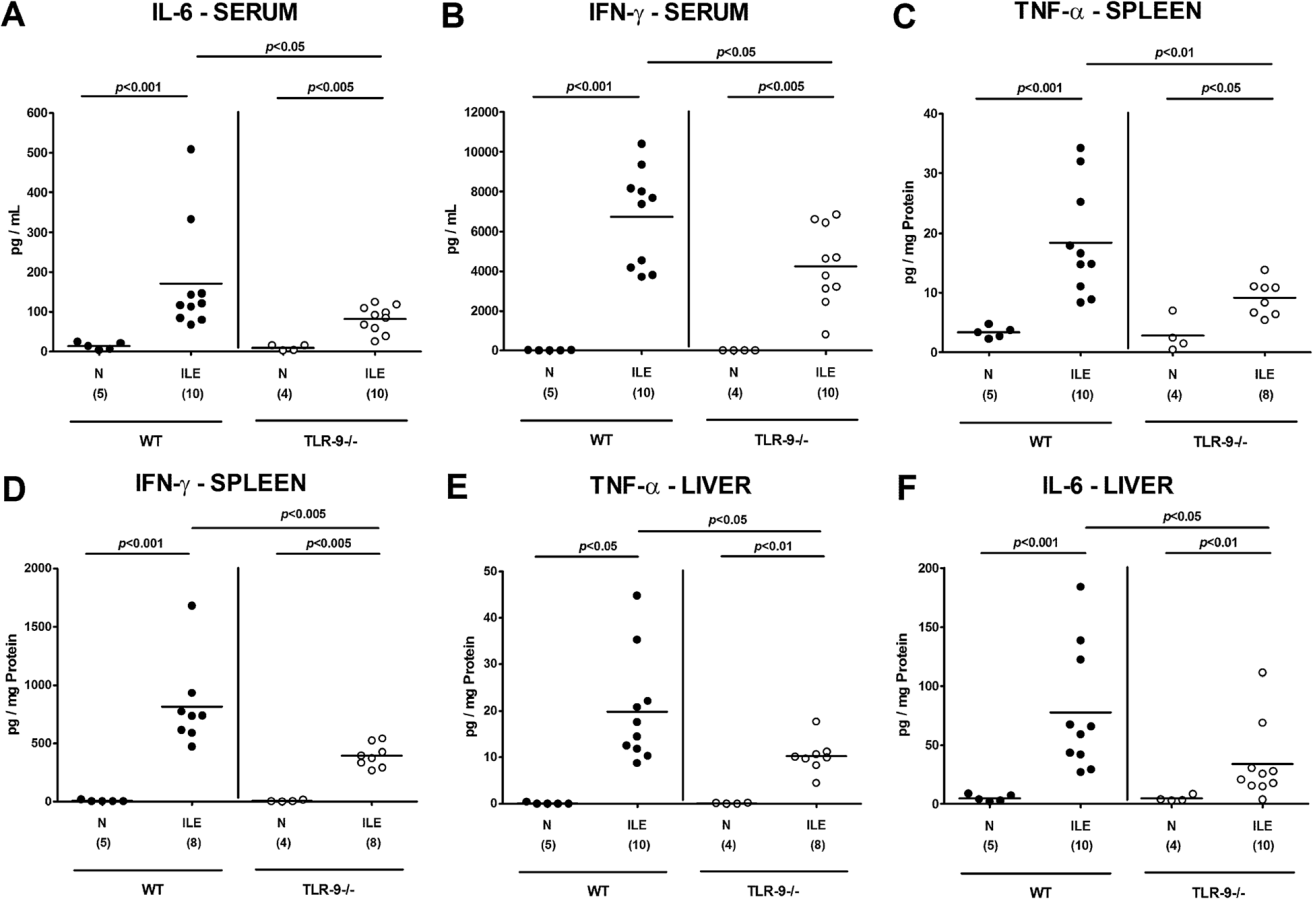
**Systemic and extra-intestinal pro-inflammatory cytokine responses following ileitis induction.** Serum **(A)** IL-6 and **(B)** IFN-γ, splenic **(C)** TNF-α and **(D)** IFN-γ as well as hepatic **(E)** TNF-α and **(F)** IL-6 protein concentrations were determined in respective *ex vivo* biopsies derived from C57BL/6 wildtype (WT; black circles) and TLR-9 deficient (TLR-9-/-; white circles) mice seven days following ileitis induction (ILE). Naïve mice served as negative controls (N). Numbers of analyzed mice (in parentheses), means (black bars) and levels of significance (*P*-values) as compared to the respective groups (determined by Mann–Whitney-*U* test) are indicated. Data shown were pooled from three independent experiments.

We next investigated systemic immune responses and potential pro-inflammatory sequelae in extra-intestinal organs following ileitis induction. Irrespective of the genotype of mice, IL-6 and IFN-γ serum levels (Figure 8A,B), splenic TNF-α and IFN-γ (Figure 8C,D) as well as hepatic TNF-α and IL-6 concentrations (Figure 8E,F) increased multifold until day 7 following infection. Remarkably, pro-inflammatory cytokine levels in the respective extra-intestinal compartment were significantly lower in TLR-9^-/-^ as compared to WT mice at day 7 p.i. (p < 0.05-0.005; Figure 8). Furthermore, TLR-9^-/-^ mice exhibited approximately 50% lower IL-6 serum concentrations as compared to WT controls at day 7 p.i. (p < 0.05; Figure 8A). Taken together, the absence of TLR-9 does not prevent the development of *T. gondii* induced acute ileitis. However, intestinal parasite loads and local immune responses are more distinct in infected TLR-9-/- as compared to WT mice. In contrast, the absence of TLR-9 results in lower levels of extra-intestinal and systemic inflammation.

**Figure 9:**
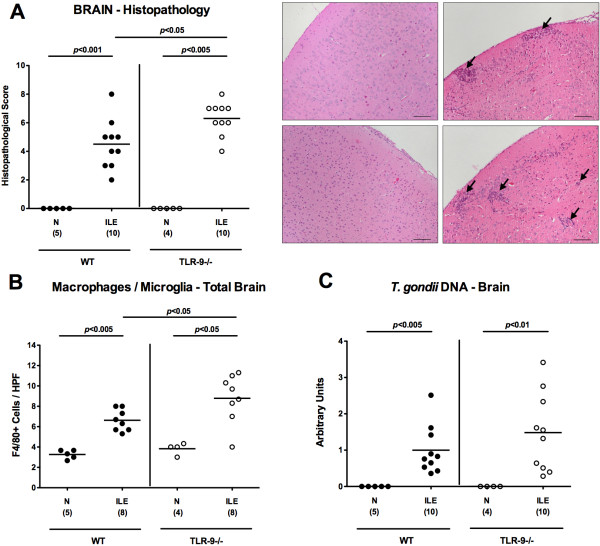
**Cerebral histopathological changes following acute ileitis induction.** C57BL/6 wildtype (WT; black circles) and TLR-9 deficient (TLR-9-/-; white circles) mice were perorally infected with 100 cysts of *T. gondii* ME49 strain on day 0 to induce acute ileitis. Naïve mice served as negative controls (N). **(A)** Cerebral histopathological changes (meninges and cortex) were assessed in H&E-stained paraffin sections of brains seven days following ileitis induction (ILE) applying a standardized histopathological score (as described in methods) and illustrated by representative photomicrographs (WT: **upper panel**, TLR-9-/-: **lower panel**; N: **left panel**, ILE: **right panel**). Arrows indicate inflammatory foci (100 × magnification, scale bar 100 μm). **(B)** Brain paraffin sections were stained for F4/80 by *in situ* immunohistochemistry and the average numbers of intracerebral macrophages and microglial cells were determined in six high power fields (HPF, 400 × magnification) per animal by light microscopy. **(C)***T. gondii* DNA loads were determined in brain homogenates by semi-quantitative RT-PCR and normalized relative to the *M. musculus ASL* gene (Arbitrary Units). Numbers of analyzed mice (in parentheses), means (black bars) and levels of significance (*P*-values) as compared to the respective groups (determined by Mann-Whitney-*U* test) are indicated. Data shown were pooled from three independent experiments.

**Figure 10:**
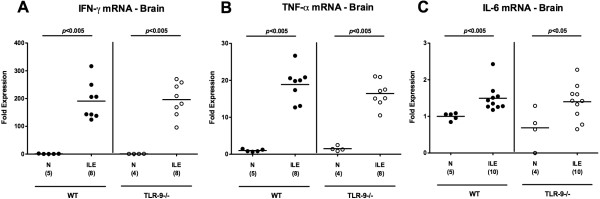
**Cerebral histopathological changes following acute ileitis induction.** C57BL/6 wildtype (WT; black circles) and TLR-9 deficient (TLR-9-/-; white circles) mice were perorally infected with 100 cysts of *T. gondii* ME49 strain on day 0 to induce acute ileitis. Naïve mice served as negative controls (N). Intracerebral **(A)** IFN-γ, **(B)** TNF-α, and **(C)** IL-6 mRNA expression levels were measured in *ex vivo* brain biopsies applying quantitative RT-PCR. Numbers of analyzed mice (in parentheses), means (black bars), and levels of significance (*P*-values) determined by Mann–Whitney-*U* test are indicated. Data shown were pooled from three independent experiments.

Next we analyzed a potential intracerebral inclusion in *T. gondii* induced acute small intestinal immunopathology. Most importantly, *T. gondii* induced acute ileitis and brain inflammation could be observed in mice of either genotype as early as day 7 p.i. (Figure 9); the cortex as well as the meninges of infected, but not naïve animals were affected (Figure 9A). These inflammatory responses, however, were more pronounced in TLR-9^-/-^ as compared to WT mice as indicated by higher histopathological scores (assessing both, cortical and meningeal inflammatory foci) in the former (p < 0.05; Figure 9A). *In situ* immunohistochemical analyses revealed higher numbers of F4/80+ inflammatory monocytes, macrophages and residential microglia in TLR-9^-/-^ as compared to WT mice (Figure 9B). Interestingly, comparable parasitic DNA loads could be detected in brain tissues derived from mice of either genotype seven days following *T. gondii* infection (Figure 9C). Cerebral IFN-γ, TNF-α, and IL-6 mRNA expression levels increased multifold upon *T. gondii* infection, but did not differ between TLR-9^-/-^ and WT mice at day 7 p.i. (Figure 10A-C). Taken together, pro-inflammatory immune responses develop not only in intestinal, but also in extra-intestinal compartments including the brain as early as 7 days after peroral infection with *T. gondii*. In the absence of TLR-9, *T. gondii* induced small intestinal and intracerebral pro-inflammatory cytokine responses are higher, whereas systemic (i.e. serum and splenic) levels are lower as compared to WT mice at day 7 p.i.

## Discussion

In the present study we show that the absence of TLR-9 does not protect mice from the development of acute ileitis following peroral infection with *T. gondii*. TLR-9^-/-^ mice succumbed to intestinal immunopathology as did WT mice. Despite a comparable clinical and intestinal (e.g. histopathological) outcome of infection we observed several TLR-9-mediated effects. For instance, TLR-9^-/-^ mice displayed higher parasitic loads in the small intestines as compared to WT mice, which was accompanied by higher levels of pro-inflammatory cytokine such as IFN-γ and NO in ilea and IFN-γ and TNF-α in MLNs in the absence of TLR-9. The *T. gondii* induced Th1 type immune response in TLR-9^-/-^ mice was further supported by a higher abundance of IFN-γ producing T cells in MLNs derived from TLR-9^-/-^ as compared to WT mice at day 7 p.i. Our results are in contrast to a previous study by Minns and colleagues demonstrating that *T. gondii* infected TLR-9^-/-^ mice were protected from disease due to a diminished Th1-type immune response [17]. TLR-9 deficiency had resulted in approximately 50% reduction of IFN-γ production in infected mice, which in turn was insufficient to combat the parasite [17]. Consequently and in agreement with our study, infected TLR-9^-/-^ mice harbored more parasites as compared to WT animals. It is unclear, however, why the overwhelming (local) Th1 response in our report was still insufficient to control parasite replication. One reason could be that a down-regulated systemic expression of pro-inflammatory cytokines as shown by lower splenic IFN-γ and TNF-α and serum IL-6 might contribute to insufficient parasite control in *T. gondii* infected TLR-9^-/-^ mice. Notably, expanding intestinal IL-10 producing FOXP3+ Treg populations exert important anti-inflammatory defense mechanisms against invading pathogens [23]. Given that WT, but not TLR-9^-/-^ mice exhibited increased numbers of ileal FOXP3+ cells upon infection, reduced Treg numbers might be indicative for compromised ileal mucosal regulatory properties in the absence of TLR-9, which in turn contributes to a more devastating inflammatory scenario. Interestingly, a previous study revealed a TLR-9 dependent induction of intestinal α-defensins comprising a conserved heterogenous group of antimicrobial peptides upon *T. gondii* infection [24]. In infected TLR-9^-/-^ mice, however, secretory granules of α-defensin producing Paneth cells failed to degranulate and, in turn, to elicit a potential local anti-parasitic effect [24]. Furthermore, we showed that in the absence of TLR-9 viable bacterial species originating from the commensal intestinal microbiota were more frequently capable of translocating through the inflamed epithelial barrier to extra-intestinal organs such as liver, spleen, kidney, and even cardiac blood. It is somewhat surprising, however, that even during more pronounced ileal disease TLR-9 deficient mice exhibited less extra-intestinal and systemic pro-inflammatory cytokine in liver, spleen, and serum as compared to WT mice at day 7 p.i.

Several factors might be responsible for the conflicting results reported by Minns et al. such as differences in *T. gondii* strain and numbers of applied cysts. Minns et al. perorally infected mice with 35 cysts of the 76 K strain, whereas in our study mice were challenged with three times more (i.e. 100) cysts of the ME49 strain. Hence, the induction of the Th1-type immunopathology was more pronounced in our present study due to the higher amount of applied *T. gondii* cysts. Furthermore, differences in the genetic background (complete versus incomplete backcrossings into the same genetic background), sex and age as well as housing conditions (e.g. diet, hygienic conditions in the animal facility) and the colonization status of the applied mice might also impact host resistance against parasitic infection [25-27]. A plethora of recent reports highlight the pivotal role of the intestinal microbiota in immunity and inflammation, and even subtle differences in the microbiota composition might have a major biological impact on the initiation and perpetuation of immunopathology [23,28-31]. This encouraged us to perform a comprehensive comparative survey of the intestinal microbiota composition in naïve and *T. gondii* infected TLR-9^-/-^ as well as WT mice. Interestingly, naïve TLR-9^-/-^ mice harbored approximately one order of magnitude lower enterobacteria (such as commensal *E. coli*) and Mouse Intestinal *Bacteroides* as compared to WT mice, whereas bifidobacteria were virtually absent in the former. One needs to take into consideration that bifidobacterial species are considered probiotic bacteria with anti-inflammatory capacities. In a previous *in vitro* study, for instance, CD4+ T cells co-cultured with bifidobacteria stimulated dendritic cells resulted in an increase of CD25+ FOXP3+ Tregs [32]. It is tempting to speculate that due to the absence of bifidobacteria in TLR-9^-/-^ mice we did not observe increased numbers of ileal FOXP3+ Treg populations upon *T. gondii* infection, which additionally contributed to a compromized host resistance against the parasite. However, it is currently virtually impossible to decipher whether the observed immune responses are mainly due to signaling of DNA signals derived from the intestinal microbiota and/or the parasite.

Experimental chronic encephalitis develops within several weeks following intraperitoneal low dose (i.e. 1 to 10 cysts) *T. gondii* infection of mice [2,33,34]. To our surprise, in the acute ileitis model presented here overt inflammatory changes were not restricted to the small intestinal tract, but could also be observed within the central nervous system affecting both, the brain cortex and meninges as early as one week following peroral high dose *T. gondii* infection in WT as well as TLR-9^-/-^ mice. Remarkably, brains of *T. gondii* infected TLR-9^-/-^ mice were even more distinctly affected by the induced pro-inflammatory immune responses as compared to WT control animals given that higher numbers of inflammatory foci and higher abundance of F4/80+ cells comprising recruited macrophages, inflammatory monocytes, and residential microglia were detectable. Interestingly, intracerebral parasitic loads and mRNA expression levels of pro-inflammatory cytokines such as IFN-γ, TNF-α, and IL-6 were similar in mice of either genotype at day 7 p.i. Even though we did not address the underlying mechanisms of this collateral damage within the hyper-acute intestinal inflammatory disease, several factors might explain the concomitant intracerebral inflammatory response. It is well described that pro-inflammatory cytokines and active molecules such as IFN-γ, TNF-α, IL-6, and reactive oxygen species are up-regulated during acute *T. gondii* infection [1,2,8,35]. TNF-α is mainly produced by Ly6C + inflammatory monocytes that regulate parasite control [35]. Importantly, systemic TNF-α has been shown to activate cerebral microglia in infection-induced encephalopathy [36] and murine neurocysticercosis [37], and subsequently up-regulates other inflammatory mediators and neurotransmitters. Furthermore, matrix-degrading mediators such as matrix metalloproteinases (MMP), e.g. gelatinases, are up-regulated in the small intestinal tract during acute *T. gondii* induced ileitis [22] and ischemic injury [38]. As a consequence, immune cells become activated [39,40] and further biologically active TNF-α and IL-6 are released from the surfaces of macrophages [25,41,42] which might lead to blood brain barrier breach and consequent influx of immune cells from the blood to the CNS [38]. Enhanced intracerebral F4/80+ monocyte recruitment might exacerbate oxidative stress to the brain parenchyma to perpetuate the inflammatory response [36].

## Conclusion

TLR-9 impacts the intestinal microbiota composition and mediates immunopathological responses during acute ileitis following peroral high dose *T. gondii* infection. Furthermore, acute *T. gondii* induced ileitis is accompanied by distinct TLR-9 dependent inflammatory sequelae in extra-intestinal compartments including the brain as early as 7 days p.i. In the absence of TLR-9 local (i.e. small intestinal) as well as intracerebral inflammatory changes are more pronounced, whereas systemic pro-inflammatory immune responses are down-regulated upon *T. gondii* infection.

## Methods

### Ethics statement

All animal experiments were conducted according to the European Guidelines for animal welfare (2010/63/EU) with approval of the commission for animal experiments headed by the “Landesamt für Gesundheit und Soziales” (LaGeSo, Berlin; registration number G0146/10). Animal welfare was monitored twice daily by assessment of clinical conditions and weight loss of mice.

### Mice and induction of acute ileitis

TLR-9^-/-^ mice (in C57BL/6 background; described elsewhere [43]) and wildtype controls were bred and housed under specific pathogen-free (SPF) conditions in the Forschungseinrichtung für Experimentelle Medizin (FEM, Charité – University Medicine Berlin, Germany). For induction of acute ileitis, age matched 3 months old female mice were infected perorally by gavage with 100 *T. gondii* cysts (ME49 strain) from homogenized brains of intraperitoneally infected NMRI mice in a volume of 0.3 mL phosphate-buffered saline (PBS), as described previously [9,10,44].

### Sampling procedures and histopathology

Mice were sacrificed by isofluran treatment (Abbott, Germany). Cardiac blood and tissue samples from brain, spleen, liver, kidneys, mesenteric lymph nodes, and ileum were removed under sterile conditions. Small intestinal samples from each mouse were collected in parallel for histopathological, immunohistochemical, microbiological, and immunological analyses. Immunohistopathological changes were determined in samples derived from ileum and brain that were immediately fixed in 5% formalin and embedded in paraffin. Sections (5 μm) were stained with hematoxylin and eosin (H&E), examined by light microscopy (magnification 100 × and 400 ×) and histopathological changes quantitatively assessed applying respective histopathological scoring systems.

**Ileal histopathology** was determined as described previously [9].

**Brain histopathology** (max. 10 points; according to [33-35] with minor modifications): The numbers of inflammatory foci per HPF (100 × magnification) were assessed in cortex and meninges (separately) and the sum of the resulting scores indicated (maximum score of 10). 0, healthy brain structure with no inflammatory foci; 1, single inflammatory foci [1-3]; 2, inflammatory foci [3-6]; 3, inflammatory foci [7-10]; 4, inflammatory foci [11-15]; 5, inflammatory foci (>15).

### Immunohistochemistry

*In situ* immunohistochemical analyses of 5 μm thin ileal paraffine sections were performed as described previously [43,45-47]. Primary antibodies against cleaved caspase-3 (Asp175, Cell Signaling, USA, 1:200), CD3 (M20, Santa Cruz, 1:1000), myeloperoxidase-7 (MPO-7, # A0398, Dako, 1:10000), F4/80 (# 14–4801, clone BM8, eBioscience, 1:50), and FOXP3 (FJK-16 s, eBioscience, 1:100) were used. For each animal, the average number of positively stained cells within at least six high power fields (HPF, 0.287 mm^2^; 400 × magnification) was determined microscopically by two independent double-blinded investigators.

### Ileal *T. gondii* DNA detection and cytokine measurement

Ileal *ex vivo* biopsies were cut longitudinally and washed in PBS. In approximately 1 cm^2^ of homogenized ileal tissue *T. gondii* DNA was measured as described previously [22]. Spleen, liver, mesenteric lymph nodes or strips of approximately 1 cm^2^ ileal tissue were placed in 24-flat-bottom well culture plates (Nunc, Wiesbaden, Germany) containing 500 μL serum-free RPMI 1640 medium supplemented with penicillin (100 U/mL) and streptomycin (100 μg/mL; PAA Laboratories). After 18 h incubation at 37°C, culture supernatants or serum samples were tested for IFN-γ, TNF-α, IL-6, and IL-10 by the Mouse Inflammation Cytometric Bead Assay (CBA; BD Biosciences) on a BD FACSCanto II flow cytometer (BD Biosciences). Nitric oxide (NO) was determined by Griess reaction as described earlier [9].

### Lymphocyte isolation, sorting and flow cytometry

Mesenteric lymph nodes were removed and subsequently minced through a 70 μm filter. For intracellular staining, cells were stimulated for 4 h with 50 ng/mL 12-O-tetradecanoylphorbol-13 acetate (Sigma, Missouri, USA), 750 ng/mL ionomycin (Sigma) and Golgi Stop (BD Biosciences, San Diego, USA) at 37°C. Stainings and cell sorting with anti-CD3 (clone 145-2C11, isotype hamster IgG1; BD Pharmingen), anti-CD4 (clone GK1.5 isotype rat IgG2b; BD Pharmingen), anti-CD69 (clone H1.2 F3, isotype Hamster IgG1*, Lamda 3; BD Pharmingen), and anti-IFN-γ (clone 145-2C11, isotype hamster IgG1; BD Pharmingen) were performed. Cells were analyzed with a FACSCalibur or LSR II Flow Cytometer (BD Biosciences).

### Molecular analysis of the intestinal microbiota

DNA from fecal samples was extracted as described previously [9]. Briefly, DNA extracts and plasmids were quantified using Quant-iT PicoGreen reagent (Invitrogen, UK) and adjusted to 1 ng per μL. Then, main bacterial groups abundant in the murine conventional intestinal microbiota were assessed by quantitative RT-PCR with group-specific 16S rRNA gene primers (Tib MolBiol, Germany) as described previously [43,45,48]. The number of 16S rRNA gene copies per ng DNA of each sample was determined and frequencies of respective bacterial groups calculated proportionally to the eubacterial (V3) amplicon.

### Bacterial translocation

For qualitative detection of bacterial translocation, entire MLNs, liver, spleen, kidneys, and cardiac blood were transferred into a thioglycolate broth each and incubated for maximum seven days at 37°C [49]. Bacterial growth was monitored daily by turbidity assessment. Aliquots of turbid broths were cultivated on respective solid media under aerobic, microaerobic, and obligate anaerobic conditions. Bacterial species identification was performed as described earlier [9].

### Cerebral cytokine and parasitic DNA detection

Brain tissue preparation and measurement of pro-inflammatory cytokine mRNA expression by quantitative real time-PCR were performed as described previously [34]. Perfused brain tissue samples were snap-frozen and kept at -80°C. 30 mg of brain tissue were used for nucleic acid purification using the spin column based AllPrep DNA/RNA/Protein Mini Kit (QIAgen, Hilden, Germany) and following the manufacturer’s instructions. On-membrane DNase I digestion (peqGOLD, Erlangen, Germany) was performed during RNA purification. RNA and DNA purity and concentration were determined by absorbance at 230, 260 and 280 nm in a NanoDrop spectrophotometer (Fisher Scientific, Germany).

Semi-quantitative real time-PCR analyses were performed to determine parasite loads in brains. FastStart Essential DNA Green Master (Roche, Grenzach-Wyhlen, Germany) was used with 90 ng genomic DNA in a reaction volume of 20 μL. Triplicate reactions were developed in a LightCycler® 480 Instrument II (Roche, Grenzach-Wyhlen, Germany). After an initial activation step (95°C for 10 min), 45 amplification cycles were run, comprising of denaturation at 95°C for 15 sec, annealing at 60°C for 15 sec and elongation at 72°C for 15 sec. The following primers were manufactured by Tib MolBiol (Berlin, Germany) and used at a final concentration of 0.3 μM: *Toxoplasma gondii* B1: (Forward) 5’- TCCCCTCTgCTggCgAAAAgT-3’ and (Reverse) 5’-AgCgTTCgTggTCAACTATCgATTg-3’ [50]. *Mus musculus* argininosuccinate lyase (ASL) gene: (Forward) 5’-TCTTCgTTAgCTggCAACTCACCT-3’ and (Reverse) 5’-ATgACCCAgCAgCTAAgCAgATCA-3’ [51]. Parasite loads (target: *Toxoplasma gondii*, B1 gene) were measured relative to mouse cell number (reference: *Mus musculus*, argininosuccinate lyase (ASL) gene), that is the target/reference ratio calculated with the LightCycler® 480 Software release 1.5.0 (Roche, Grenzach-Wyhlen, Germany).

To determine relative gene expression, SuperScript® III Platinum® One-Step Quantitative RT-PCR System (life technologies, Darmstadt, Germany) was used with 300 ng total RNA in a reaction volume of 10 μL. Triplicate reactions were developed in a LightCycler® 480 Instrument II (Roche, Grenzach-Wyhlen, Germany). Reverse transcription was performed for 15 min at 50°C followed by 2 min at 95°C. Subsequently, 45 amplification cycles were run, comprising of denaturation at 95°C for 15 sec and annealing/elongation at 60°C for 30 sec. TaqMan® Gene Expression Assays (life technologies, Darmstadt, Germany) were used for amplification of the house-keeping gene HPRT, IFN-γ TNF-α, and IL-6. HPRT expression was chosen as reference for normalization and target/reference ratios were calculated with the LightCycler® 480 Software release 1.5.0 (Roche, Grenzach-Wyhlen, Germany). Resulting data were further normalized on values of control groups.

### Statistical analysis

Medians, means and levels of significance were determined using Mann–Whitney *U*-Test. Two-sided probability (*P*) values ≤0.05 were considered significant. All experiments were performed three times.

## Competing interests

The authors have declared that no competing interests exist.

## Authors’ contributions

Conceived and designed the experiments: IRD, MMH. Performed the experiments: AAK, MA, AF, LM, DS, IRD, MMH. Analyzed the data: AAK, AF, LM, DS, OL, IRD, MMH. Contributed reagents/materials/analysis tolls: SB, AAK, OL, UBG. Wrote the paper: SB, AAK, AF, IRD, MMH. All authors read and approved the final manuscript.

## Authors’ information

Ildikò R Dunay and Markus M Heimesaat shared last authorship.

## References

[B1] Liesenfeld O Kosek J Remington JS Suzuki Y Association of CD4+ T cell-dependent, interferon-gamma-mediated necrosis of the small intestine with genetic susceptibility of mice to peroral infection with *Toxoplasma gondii* J Exp Med 1996 184 597 607 10.1084/jem.184.2.597 8760813 PMC2192709

[B2] Munoz M Liesenfeld O Heimesaat MM Immunology of *Toxoplasma gondii* Immunol Rev 2011 240 269 285 10.1111/j.1600-065X.2010.00992.x 21349099

[B3] Khan IA Schwartzman JD Matsuura T Kasper LH A dichotomous role for nitric oxide during acute *Toxoplasma gondii* infection in mice Proc Natl Acad Sci U S A 1996 94 13955 13960 9391134 10.1073/pnas.94.25.13955 PMC28414

[B4] Liesenfeld O Kang H Park D Nguyen TA Parkhe CV Watanabe H Abo T Sher A Remington JS Suzuki Y TNF-alpha, nitric oxide and IFN-gamma are all critical for development of necrosis in the small intestine and early mortality in genetically susceptible mice infected perorally with *Toxoplasma gondii* Parasite Immunol 1999 21 365 376 10.1046/j.1365-3024.1999.00237.x 10417671

[B5] Mennechet FJL Kasper H Rachinel N Li W Vandewalle A Buzoni-Gatel D Lamina propria CD4+ T lymphocytes synergize with murine intestinal epithelial cells to enhance proinflammatory response against an intracellular pathogen J Immunol 2002 168 2988 2996 10.4049/jimmunol.168.6.2988 11884471

[B6] Vossenkamper A Struck D Alvarado-Esquivel C Went T Takeda K Akira S Pfeffer K Alber G Lochner M Forster I Liesenfeld O Both IL-12 and IL-18 contribute to small intestinal Th1-type immunopathology following oral infection with *Toxoplasma gondii*, but IL-12 is dominant over IL-18 in parasite control Eur J Immunol 2004 34 3197 3207 10.1002/eji.200424993 15368276

[B7] Buzoni-Gatel D Schulthess J Menard LC Kasper LH Mucosal defences against orally acquired protozoan parasites, emphasis on *Toxoplasma gondii* infections Cell Microbiol 2006 8 535 544 10.1111/j.1462-5822.2006.00692.x 16548880

[B8] Liesenfeld O Oral infection of C57BL/6 mice with *Toxoplasma gondii*: a new model of inflammatory bowel disease? J Infect Dis 2002 185 Suppl 1 S96 S101 11865446 10.1086/338006

[B9] Heimesaat MM Bereswill S Fischer A Fuchs D Struck D Niebergall J Jahn HK Dunay IR Moter A Gescher DM Schumann RR Göbel UB Liesenfeld O Gram-negative bacteria aggravate murine small intestinal Th1-type immunopathology following oral infection with *Toxoplasma gondii* J Immunol 2006 177 8785 8795 10.4049/jimmunol.177.12.8785 17142781

[B10] Heimesaat MM Fischer A Jahn HK Niebergall J Freudenberg JM Blaut M Liesenfeld O Schumann RR Göbel UB Bereswill S Exacerbation of murine ileitis by Toll-like receptor 4 mediated sensing of lipopolysaccharide from commensal *Escherichia coli* Gut 2007 56 941 948 10.1136/gut.2006.104497 17255219 PMC1994376

[B11] Erridge C Duncan SH Bereswill S Heimesaat MM The induction of colitis and ileitis in mice is associated with marked increases in intestinal concentrations of stimulants of TLRs 2, 4, and 5 PLoS One 2011 5 e9125 20161736 10.1371/journal.pone.0009125 PMC2817728

[B12] Hemmi H Takeuchi O Kawai T Kaisho T Sato S Sanjo H Matsumoto M Hoshino K Wagner H Takeda K Akira S A Toll-like receptor recognizes bacterial DNA Nature 2000 408 740 745 10.1038/35047123 11130078

[B13] Takeda K Akira S Toll-like receptors in innate immunity Int Immunol 2005 17 1 14 15585605 10.1093/intimm/dxh186

[B14] Rutz M Metzger J Gellert T Luppa P Lipford GB Wagner H Bauer S Toll-like receptor 9 binds single-stranded CpG-DNA in a sequence- and pH-dependent manner Eur J Immunol 2004 34 2541 2550 10.1002/eji.200425218 15307186

[B15] Honda K Yanai H Mizutani T Negishi H Shimada N Suzuki N Ohba Y Takaoka A Yeh WC Taniguchi T Role of a transductional-transcriptional processor complex involving MyD88 and IRF-7 in Toll-like receptor signaling Proc Natl Acad Sci U S A 2004 101 15416 15421 10.1073/pnas.0406933101 15492225 PMC523464

[B16] Kim JM Kim NI Oh YK Kim YJ Youn J Ahn MJ CpG oligodeoxynucleotides induce IL-8 expression in CD34+ cells via mitogen-activated protein kinase-dependent and NF-kappaB-independent pathways Int Immunol 2005 17 1525 1531 10.1093/intimm/dxh345 16263754

[B17] Minns LA Menard LC Foureau DM Darche S Ronet C Mielcarz DW Buzoni-Gatel D Kasper LH TLR9 is required for the gut-associated lymphoid tissue response following oral infection of *Toxoplasma gondii* J Immunol 2006 176 7589 7597 10.4049/jimmunol.176.12.7589 16751405

[B18] Yarovinsky F Innate immunity to *Toxoplasma gondii* infection Nat Rev Immunol 2014 14 109 121 10.1038/nri3598 24457485

[B19] Andrade WA Souza Mdo C Ramos-Martinez E Nagpal K Dutra MS Melo MB Bartholomeu DC Ghosh S Golenbock DT Gazzinelli RT Combined action of nucleic acid-sensing Toll-like receptors and TLR11/TLR12 heterodimers imparts resistance to *Toxoplasma gondii* in mice Cell Host Microbe 2013 13 42 53 10.1016/j.chom.2012.12.003 23290966 PMC3552114

[B20] Benson A Pifer R Behrendt CL Hooper LV Yarovinsky F Gut commensal bacteria direct a protective immune response against *Toxoplasma gondii* Cell Host Microbe 2009 6 187 196 10.1016/j.chom.2009.06.005 19683684 PMC2746820

[B21] Suzuki Y Sher A Yap G Park D Neyer LE Liesenfeld O Fort M Kang H Gufwoli E IL-10 is required for prevention of necrosis in the small intestine and mortality in both genetically resistant BALB/c and susceptible C57BL/6 mice following peroral infection with *Toxoplasma gondii* J Immunol 2000 164 5375 5382 10.4049/jimmunol.164.10.5375 10799901

[B22] Munoz M Heimesaat MM Danker K Struck D Lohmann U Plickert R Bereswill S Fischer A Dunay IR Wolk K Loddenkemper C Krell HW Libert C Lund LR Frey O Holscher C Iwakura Y Ghilardi N Ouyang W Kamradt T Sabat R Liesenfeld O Interleukin (IL)-23 mediates *Toxoplasma gondii*-induced immunopathology in the gut via matrixmetalloproteinase-2 and IL-22 but independent of IL-17 J Exp Med 2009 206 3047 3059 10.1084/jem.20090900 19995958 PMC2806449

[B23] Belkaid Y Hand TW Role of the microbiota in immunity and inflammation Cell 2014 157 121 141 10.1016/j.cell.2014.03.011 24679531 PMC4056765

[B24] Foureau DM Mielcarz DW Menard LC Schulthess J Werts C Vasseur V Ryffel B Kasper LH Buzoni-Gatel D TLR9-dependent induction of intestinal alpha-defensins by *Toxoplasma gondii* J Immunol 2010 184 7022 7029 10.4049/jimmunol.0901642 20488791

[B25] Heimesaat MM Dunay IR Fuchs D Trautmann D Fischer A Kühl AA Loddenkemper C Siegmund B Batra A Bereswill S Liesenfeld O The distinct roles of MMP-2 and MMP-9 in acute DSS colitis Eur J Microbiol Immunol (Bp) 2011 1 302 310 10.1556/EuJMI.1.2011.4.6 24516737 PMC3918133

[B26] Deloris Alexander A Orcutt RP Henry JC Baker JJR Bissahoyo AC Threadgill DW Quantitative PCR assays for mouse enteric flora reveal strain-dependent differences in composition that are influenced by the microenvironment Mamm Genome 2006 17 1093 1104 10.1007/s00335-006-0063-1 17091319

[B27] Ge Z Feng Y Taylor NS Ohtani M Polz MF Schauer DB Fox JG Colonization dynamics of altered Schaedler flora is influenced by gender, aging, and *Helicobacter hepaticus* infection in the intestines of Swiss Webster mice Appl Environ Microbiol 2006 72 5100 5103 10.1128/AEM.01934-05 16820515 PMC1489328

[B28] Sydora BC Macfarlane SM Walker JW Dmytrash AL Churchill TA Doyle J Fedorak RN Epithelial barrier disruption allows nondisease-causing bacteria to initiate and sustain IBD in the IL-10 gene-deficient mouse Inflamm Bowel Dis 2007 13 947 954 10.1002/ibd.20155 17427241

[B29] Bloom SM Bijanki VN Nava GM Sun L Malvin NP Donermeyer DL Dunne WMJR Allen PM Stappenbeck TS Commensal *Bacteroides* species induce colitis in host-genotype-specific fashion in a mouse model of inflammatory bowel disease Cell Host Microbe 2011 9 390 403 10.1016/j.chom.2011.04.009 21575910 PMC3241010

[B30] Sartor RB Microbial influences in inflammatory bowel diseases Gastroenterology 2008 134 577 594 10.1053/j.gastro.2007.11.059 18242222

[B31] Sartor RB Genetics and environmental interactions shape the intestinal microbiome to promote inflammatory bowel disease versus mucosal homeostasis Gastroenterology 2010 139 1816 1819 10.1053/j.gastro.2010.10.036 21029802

[B32] O’Mahony D Murphy S Boileau T Park J O’Brien F Groeger D Konieczna P Ziegler M Scully Shanahan PF Kiely B O’Mahony L *Bifidobacterium animalis* AHC7 protects against pathogen-induced NF-kappaB activation in vivo BMC Immunol 2010 11 63 10.1186/1471-2172-11-63 21176205 PMC3016395

[B33] Dunay IR Heimesaat MM Bushrab FN Muller RH Stocker H Arasteh K Kurowski M Fitzner R Borner K Liesenfeld O Atovaquone maintenance therapy prevents reactivation of toxoplasmic encephalitis in a murine model of reactivated toxoplasmosis Antimicrob Agents Chemother 2004 48 4848 4854 10.1128/AAC.48.12.4848-4854.2004 15561866 PMC529229

[B34] Mohle L Parlog A Pahnke J Dunay IR Spinal cord pathology in chronic experimental Toxoplasma gondii infection Eur J Microbiol Immunol (Bp) 2014 4 65 75 10.1556/EuJMI.4.2014.1.6 24678407 PMC3955833

[B35] Dunay IR Damatta A Fux B Presti R Greco S Colonna M Sibley LD Gr1 (+) inflammatory monocytes are required for mucosal resistance to the pathogen Toxoplasma gondii Immunity 2008 29 306 317 10.1016/j.immuni.2008.05.019 18691912 PMC2605393

[B36] Young GB Encephalopathy of infection and systemic inflammation J Clin Neurophysiol 2013 30 454 461 10.1097/WNP.0b013e3182a73d83 24084178

[B37] Mishra BB Mishra PK Teale JM Expression and distribution of Toll-like receptors in the brain during murine neurocysticercosis J Neuroimmunol 2006 181 46 56 10.1016/j.jneuroim.2006.07.019 17011049 PMC1779953

[B38] Carty M Bowie AG Evaluating the role of Toll-like receptors in diseases of the central nervous system Biochem Pharmacol 2011 81 825 837 10.1016/j.bcp.2011.01.003 21241665

[B39] Basset C Holton J Inflammatory bowel disease: is the intestine a Trojan horse? Sci Prog 2002 85 33 56 10.3184/003685002783238861 11969118 PMC10361205

[B40] Martinesi M Treves C Bonanomi AG Milla M Bagnoli S Zuegel Steinmeyer UA Stio M Down-regulation of adhesion molecules and matrix metalloproteinases by ZK 156979 in inflammatory bowel diseases Clin Immunol 2010 136 51 60 10.1016/j.clim.2010.03.004 20399147

[B41] Naito Y Takagi T Kuroda M Katada K Ichikawa H Kokura S Yoshida N Okanoue NT Yoshikawa T An orally active matrix metalloproteinase inhibitor, ONO-4817, reduces dextran sulfate sodium-induced colitis in mice Inflamm Res 2004 53 462 468 10.1007/s00011-004-1281-1 15550999

[B42] Wang M Qin X Mudgett JS Ferguson TA Senior RM Welgus HG Matrix metalloproteinase deficiencies affect contact hypersensitivity: stromelysin-1 deficiency prevents the response and gelatinase B deficiency prolongs the response Proc Natl Acad Sci U S A 1999 96 6885 6889 10.1073/pnas.96.12.6885 10359808 PMC22011

[B43] Heimesaat MM Nogai A Bereswill S Plickert R Fischer A Loddenkemper C Steinhoff U Tchaptchet S Thiel E Freudenberg MA Göbel UB Uharek L MyD88/TLR9 mediated immunopathology and gut microbiota dynamics in a novel murine model of intestinal graft-versus-host disease Gut 2010 59 1079 1087 10.1136/gut.2009.197434 20639251

[B44] Heimesaat MM Plickert R Fischer A Göbel UB Bereswill S Can microbiota transplantation abrogate murine colonization resistance against *Campylobacter jejuni*? Eur J Microbiol Immunol (Bp) 2013 3 36 43 10.1556/EuJMI.3.2013.1.5 24265916 PMC3832074

[B45] Bereswill S Fischer A Plickert R Haag LM Otto B Kühl AA Dashti JL Zautner AE Munoz M Loddenkemper C Gross U Göbel UB Heimesaat MM Novel murine infection models provide deep insights into the “Menage a Trois” of *Campylobacter jejuni,* microbiota and host innate immunity PLoS One 2011 6 e20953 10.1371/journal.pone.0020953 21698299 PMC3115961

[B46] Haag LM Fischer A Otto B Plickert R Kühl AA Göbel UB Bereswill S Heimesaat MM *Campylobacter jejuni* induces acute enterocolitis in gnotobiotic IL-10-/- mice via Toll-like-receptor-2 and -4 signaling PLoS One 2012 7 e40761 10.1371/journal.pone.0040761 22808254 PMC3393706

[B47] Heimesaat MM Haag LM Fischer A Otto B Kühl AA Göbel UB Bereswill S Survey of extra-intestinal immune responses in asymptomatic long-term *Campylobacter jejuni*-infected mice Eur J Microbiol Immunol (Bp) 2013 3 174 182 10.1556/EuJMI.3.2013.3.4 24265935 PMC3832099

[B48] Rausch S Held J Fischer A Heimesaat MM Kühl AA Bereswill S Hartmann S Small intestinal nematode infection of mice is associated with increased enterobacterial loads alongside the intestinal tract PLoS One 2013 8 e74026 10.1371/journal.pone.0074026 24040152 PMC3769368

[B49] Heimesaat MM Boelke S Fischer A Haag LM Loddenkemper C Kühl AA Göbel UB Bereswill S Comprehensive postmortem analyses of intestinal microbiota changes and bacterial translocation in human flora associated mice PLoS One 2012 7 e40758 10.1371/journal.pone.0040758 22808253 PMC3395637

[B50] Wilson EH Wille-Reece U Dzierszinski F Hunter CA A critical role for IL-10 in limiting inflammation during toxoplasmic encephalitis J Neuroimmunol 2005 165 63 74 10.1016/j.jneuroim.2005.04.018 16005735

[B51] Butcher BA Fox BA Rommereim LM Kim SG Maure KJ Yarovinsky F Herbert DR Bzik DJ Denker EY *Toxoplasma gondii* rhoptry kinase ROP16 activates STAT3 and STAT6 resulting in cytokine inhibition and arginase-1-dependent growth control PLoS Pathog 2011 7 e1002236 10.1371/journal.ppat.1002236 21931552 PMC3169547

